# Inability to detect significant absorption of immunoreactive soya protein in healthy adults may be relevant to its weak allergenicity

**DOI:** 10.1186/2045-7022-3-6

**Published:** 2013-02-04

**Authors:** Cecilia M Lund, Christina G Dirks, Mona H Pedersen, Bettina M Jensen, Lars K Poulsen

**Affiliations:** 1 Laboratory of Medical Allergology, Allergy Clinic, Copenhagen University Hospital, Gentofte, Department 22, Niels Andersen Vej 65, Hellerup, DK-2900, Denmark

**Keywords:** Soya protein, Food allergen, Absorption, Histamine release

## Abstract

Soya and peanut are botanically closely related and share cross-reacting antigens, but compared to soya, peanut allergy has a higher prevalence with more severe allergic reactions. Furthermore, the threshold dose for eliciting reactions is higher for soya. A difference in undigested protein absorption between the two foods, might explain this diversity.

In the current study the amount of soya protein absorbed after soya bean ingestion in healthy adults was estimated. Ten subjects ingested 100 grams of soya beans (40 grams of soya protein) and blood was drawn before and 1, 3 and 24 hours after administration. Serum was analysed by ELISA and histamine release (HR). In all serum samples the soya protein concentration was below quantification limit (1.6 ng/ml which corresponds to 4.8 μg or 0.12 parts per million absorbed soya protein.

We could not detect any significant absorption of soya protein. While we cannot totally exlude technical reasons, it may also reflect a true poor absorption in healthy adult volunteers. This could, in turn, be relevant to the apparently weak allergenicity of soy protein by comparison with peanut protein in allergic subjects.

## 

Soya is frequently used as protein enrichment, and the human exposure is therefore as widespread as for peanut. Food allergy to the legumes peanut and soya display, however, quite different prevalence, natural history and severity, in spite of sharing antigenic fractions [1]. Soya generally gives a transient allergy in childhood, with very few anaphylactic and fatal reactions throughout the world whereas peanut allergy causes acute reactions with respiratory problems, skin- and gastrointestinal symptoms [2]. IgE antibodies to both foods are commonly found in individuals clinically reacting to either of them [3]. However, a surprisingly low rate of clinical co-reactivity between peanut and soya is reported. Among 75 peanut-allergic children, none had a history of soya allergy and though 58% had IgE to soya, only 2/22 patients had a positive oral challenge [4]. This might correlate with the threshold dosages for eliciting a reaction in 1% of food allergic patients where 2.7 mg peanut but 295 mg soya flour was estimated [5].

These highly varying thresholds could reflect differences in absorption or distribution of the two proteins. We have previously investigated the absorption of peanut [6], where 17 non-allergic subjects ingested 5-100 g of peanuts. Immunoreactive peanut protein in serum, were determined by ELISA and histamine release (HR) and compatible kinetics were found with protein being detectible 10-30 min after ingestion and peaking at 2-3 hours. No such investigations have been performed for soya protein.

We aimed to determine the absorption of soya, i.e. immunoreactive and potentially allergenic protein in serum of healthy adults after ingesting soya beans. Raw soya beans were considered to be uneatable and accordingly a meal of cooked soya beans was considered the optimal source of intact soya allergens.

### Findings

#### Materials and methods

Ten subjects (aged 21-28, 5 females) without history of any type of allergy, atopic dermatitis, rhinitis, or asthma were included. Pregnancy, daily medication (except birth-control) and significant concurrent disease were exclusion criteria. Absence of allergy was confirmed by a negative blood screening for peanut and soya bean IgE (Phadia, Uppsala, Sweden) and a negative skin prick test, performed in accordance with guidelines from European Academy of Allergy and Clinical Immunogy. In the skin prick test, the subjects were tested with the standard panel of inhalant allergens (ALK-Abello Hørsholm, Denmark), soya bean (cooked, raw and powder) and peanut prick-prick tests. The study was approved by the local ethical committee (j. nr. KF 01-081/01).

In a single dose soya bean challenge the subjects were given 100 g of dry soya beans, i.e. 40 g of protein. The ecological soya beans with a declared content of 40% of protein, 27% of carbohydrates and 18% of fat, soaked in water for 16 hours at 5°C and were then boiled in water for 60 minutes. The soya beans were served in a canned tomato sauce with garlic, basil, 1 tsp sugar and salt. After abstaining for 24 hours from soya products and 8 hours of fasting, the meal was ingested. Blood samples were drawn before and 1, 3 and 24 hours after the beginning of the meal. Serum was collected and stored at -20°C.

The soya protein ELISA was a direct sandwich assay employing rabbit anti-soya antibodies against an aqueous extract of unprocessed soya beans [7]. The standard curve was performed with the same extract (0.8 ng/ml - 600 ng/ml) (protein determined by amino acid analysis), and the limit of detection was determined as the background + 3 x S.D. (Figure 1).

**Figure 1:**
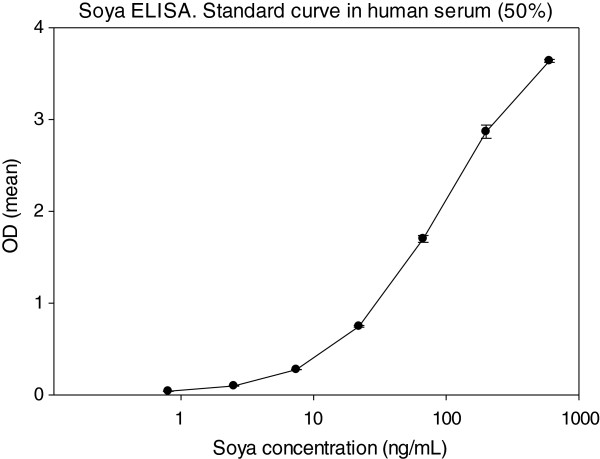
**Standard curve in the soya protein ELISA using a 50% dilution of normal human serum as diluent.** Soya protein was diluted 3-fold from 600 ng/ml down to 0.8 ng/ml. In this particular experiment, the O.D. of 0.8 ng/ml was 0.041 (S.D. = 0.001) and that of the blank was 0.025 (0.004). The O.D. of blank, where normal buffer was used, was 0.013 (0.002) (n = 24).

For determination of soya allergens by histamine release technique, human basophils in peripheral blood mononuclear cells were passively sensitized with soya specific IgE (serum from severe soya allergic) or non-specific IgE (serum from healthy control) [7]. Cells were challenged (1 hour) with serum (20% final concentration) from the 10 subjects. A standard curve (25 pg/ml – 80 ng/ml) was made with soya bean extract (Greer, NC, USA) in 20% control serum. Supernatant was analysed fluorometrically for histamine using glass fiber coated micro titer plates as described in Stahl Skov et al [8]. Results are expressed as percentage of total cellular histamine content (% HR).

### Results

Using the soya protein ELISA, which was done in duplicate, we could only repeatedly detect a weak signal in a single sample (subject F, 24 h) (Figure 2), having an OD 0.06 (0 ng + 3 x SD = 0.023). Using HR, which also was performed in duplicate, a significant response was again observed with the same sample, however with a concentration below the last point of the standard curve. Accordingly the concentration of soya protein was estimated as <1.6 ng/ml (ELISA, 1 + 1 dilution) and < 25 pg/ml (HR).

**Figure 2:**
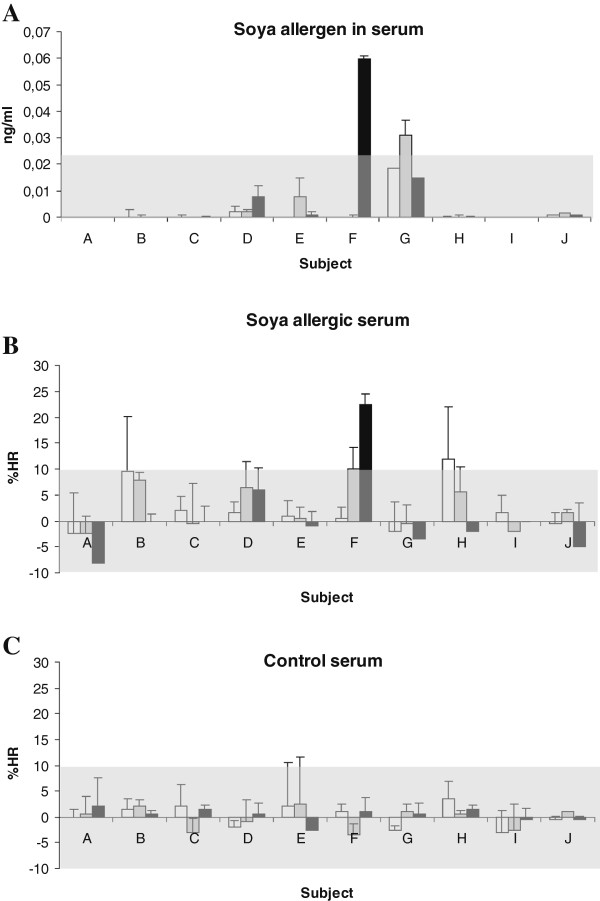
**Detection of soya allergen in serum from soya challenged subjects. A**) Serum samples (0, 1, 3 and 24 hour after ingestion of soya meal) were analyzed by ELISA. Results were corrected from background (0 hour serum sample) and samples with an OD > 0.023 ng/ml (mean + 3 x SD, 0 h samples) were considered positive for soya. Gray zone indicate cut off value. **B**) Human basophils (n = 2 donors) sensitized with IgE from soya allergic or healthy control were stimulated with serum from the challenged subjects (0, 1, 3 and 24 hour after ingestion of soya meal). Results (% released histamine,%HR) were corrected from background (0 hour serum sample) and samples with a%HR > 9.3% (mean + 3 x SD, control serum) were considered positive for soya. Gray zones indicate cut off value. White square: 1 hour, Gray square: 3 hour, Black square: 24 hour.

### Discussion

Using two different assays, of which one was known to identify whole intact proteins [7] and the other based on IgE and thus detecting allergens, we could only marginally detect soya protein in one serum sample (24 hour). There were only 10 subjects in this study. But since our results were uniformly negative for all 10 subjects, (in addition to 3 pilot subjects treated with different doses) we do not think that adding more subjects would substantially change the conclusions.

Low soya absorption could be caused by low protein dose or destruction during processing or digestion. In a pilot project 3 subjects were served 150 g of soya beans, but all were unable to eat more than a 100 g, which also was the highest intake in the peanut study. Since the general protein content of peanut is about 25% compared to 40% in soya bean, the ingested amount of protein is higher in this study. Furthermore, soya beans were cooked for 60 minutes to make them eatable, however, this treatment is found not to change IgE and IgG epitopes [9].

Some proteins are known to be rapidly degraded at pH 2.0, imitating gastric acid [10]. There are no such human data for soya protein, but studies in pigs suggest that the major storeage protein glycinin and con-glycining, which are important allergens in humans, [11] maintain some immunogenicity after passing the stomach [12].

In our peanut absorption study [6], protein concentrations varied between 4-15 ng/ml, with a peak after 2-3 hours and detectable peanut protein after 24 h. However, absorbed protein showed a considerably inter-individual variation. Both studies were performed on healthy adults, and it would be interesting to investigate if there is a difference in absorption of proteins in healthy persons compared to allergic patients. Husby *et al*. [13] observed concentrations of 10.5 ng/ml ovalbumin in blood, 3 hours after ingestion of 3 g of ovalbumin. Castell *et al.*[14] found protein concentrations of 2000-10000 pg in blood 48 h after ingestion of bromelain, 4 g/day. The absorption described in these three studies plus our own peanut study is far higher than in the present soya study, where the absolute amount in plasma was below 4.8 μg or 0.12 parts per million (ppm) of the 40 g of soya protein consumed.

These results support our theory of soya being a poorly absorbed protein, which may explain its moderate allergenicity in comparison with the high allergenicity of peanut.

### Abbreviations

HR: Histamine release.

### Competing interests

The authors have no competing interests.

### Authors’ contributions

CML carried out the clinical study, recruitment of study population, data acquisition and analysis, designed the case report forms and drafted the manuscript. BMJ was responsible for the conduction of the histamine release test, MHP was responsible for the conduction of the ELISA measurements. CGD participated in coordination of the clinical study and manuscript revising. LKP designed the study, wrote the protocol and participated in data analysis and manuscript revising. All authors have read and approved the final manuscript.
